# Inspirations of Biomimetic Affinity Ligands: A Review

**DOI:** 10.1021/acsomega.2c03530

**Published:** 2022-09-09

**Authors:** Aykut Arif Topçu, Seçkin Kılıç, Erdoğan Özgür, Deniz Türkmen, Adil Denizli

**Affiliations:** † Medical Laboratory Program, Vocational School of Health Service, Aksaray University, 68100 Aksaray, Turkey; ‡ Department of Chemistry, Hacettepe University, 06230 Ankara, Turkey

## Abstract

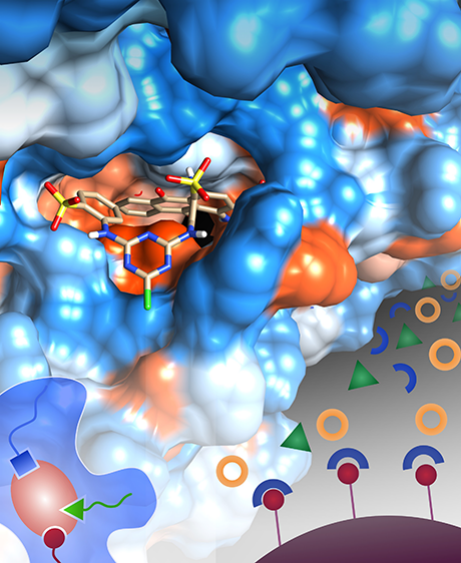

Affinity chromatography is a well-known method dependent on molecular recognition and is used to purify biomolecules by mimicking the specific interactions between the biomolecules and their substrates. Enzyme substrates, cofactors, antigens, and inhibitors are generally utilized as bioligands in affinity chromatography. However, their cost, instability, and leakage problems are the main drawbacks of these bioligands. Biomimetic affinity ligands can recognize their target molecules with high selectivity. Their cost-effectiveness and chemical and biological stabilities make these antibody analogs favorable candidates for affinity chromatography applications. Biomimetics applies to nature and aims to develop nanodevices, processes, and nanomaterials. Today, biomimetics provides a design approach to the biomimetic affinity ligands with the aid of computational methods, rational design, and other approaches to meet the requirements of the bioligands and improve the downstream process. This review highlighted the recent trends in designing biomimetic affinity ligands and summarized their binding interactions with the target molecules with computational approaches.

## 1 Introduction

Affinity chromatography is a potent and highly selective separation method for isolating biomolecules from crude samples and depends on reversible and specific interactions between the affinity ligands and biomolecules.^1^ Wilchek et al.^2^ reported the initial studies of affinity chromatography, who purified the enzymes using their substrates and their inhibitors as affinity ligands.^2^

Nowadays, this well-established separation technique is also adapted in various fields such as biosensing, drug delivery systems, and tissue engineering studies.^3^

**Figure 1:**
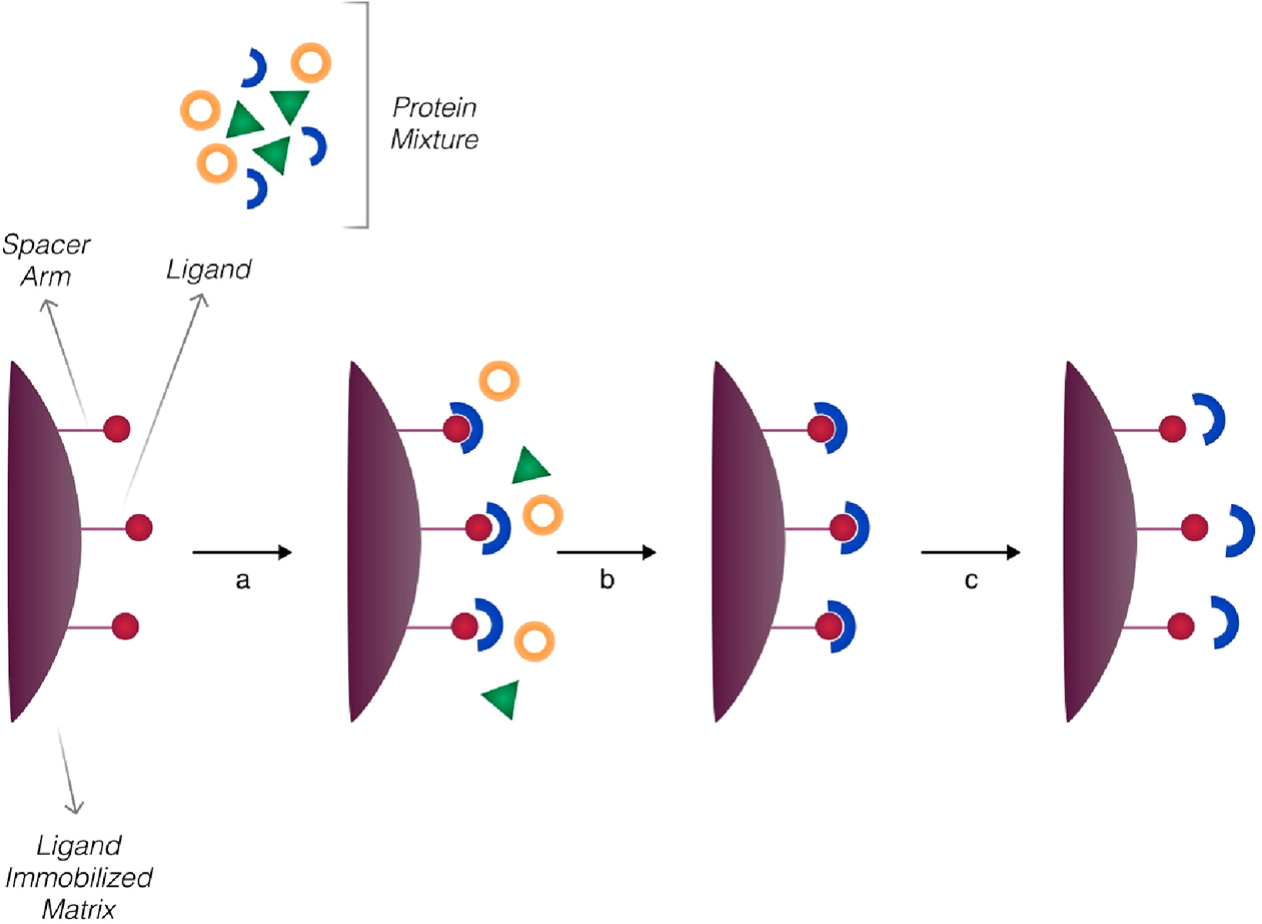
Schematic diagram of affinity chromatography: (a) loading, (b) capture of a target molecule, and (c) elution of the target molecule.

Figure 1 shows the schematic diagram of affinity chromatography. In the first step, the molecule referred to as ligand is mostly covalently immobilized onto a support material via a spacer arm. The mixture containing the target molecule is loaded on the affinity column. Finally, the captured molecule on the column is eluted by adjusting pH, ionic strength, and temperature.

Biorecognition of the target molecule plays a crucial role in affinity chromatography applications. The ligand selection is essential for capturing the target molecule from the complex media.

From this point of view, affinity ligands are classified as biospecific ligands (antigen–antibody, lectin-glycoprotein) and pseudospecific ligands, including synthetic ligands (dyes, metal-chelators) and biomimetic ligands (peptides and triazine-based ligands).^4^ Biospecific ligands are particular and selective toward the target molecules; however, their high cost, instabilities, and leakage problems are some drawbacks.

Biomimetic ligands can be used as alternative affinity ligands instead of their natural counterparts due to mimicking the critical residues that play a significant role in the recognition process.^4^ Moreover, these synthetic ligands are low-cost, have low immunogenicity, and have higher chemical stability than natural affinity ligands.^4^ These biomimetic affinity ligands have recently been designed using advanced methods such as computer-based screening technology and combinatorial technology.^5^ Hence, the development of new methods and biomimetic approaches allows for designing the appropriate biomimetic affinity ligands to purify different target molecules and wide applications.

## 2 Basic Concepts of Biomimetics

Biomimetics is an interdisciplinary field including natural sciences, engineering, and materials sciences. It mimics nature or biological systems to develop nanomaterials, nanodevices, and processes.^6^ The history of biomimetics dates back to the existence of humans, and by observing and imitating nature, humanity succeeded in designing flying machines, battleships, and powered airplanes in the 1900s.^7^ However, the term biomimetics was coined by Schmitt in 1957 during his doctoral studies, who developed a physical machine that mimics the electrical activity of a nerve.^1^ Later, in 1960, Jack E. Steele coined the word bionically. The term biomimetic was defined in a paper in 1969, which led to the introduction of the biomimetic word into the dictionary in 1974.^2^ Consequently, biomimetic studies have been taken a step further and have taken their place in the literature.^8^

Nowadays, biomimetics aims to develop nanomaterials, processes, and nanodevices by combining technology and imitating nature to save human lives and enhance life qualities.^6^

During the initial studies of biomimetic affinity chromatography, metal-chelates^9,10^ and dyes^11,12^ were used as biomimetic affinity ligands; however, these biomimetic ligands lacked selectivity against the target molecules. Recently, biomimetic ligands with high selectivity and specificity have been designed using computer simulation,^13^ combinatorial chemistry,^14^ and crystallization technique^15^ for protein purification studies.

## 3 Computational Approach for Ligand Evaluation

Molecular docking is a computational approach to predicting the experimental binding mode and affinity of a ligand that binds to the active sites of the receptor.^16^ Subsequently, using a scoring function for molecular docking makes it possible to predict the binding free energy, the binding affinity, and the binding constant of the complexes.^17^ A practical molecular docking approach necessitates a structural data bank and a method for ligand evaluation. Ultimately, several ligand poses are accepted or rejected according to the scoring function of the docking software.^17^

Computer simulation and shape complementarity approaches are widely used methods for molecular docking to design new ligands with more specificity and better efficacy toward the target of interest.^17^

During the computer simulation approach, first, the ligand and the target molecule are separated by physical distance after the ligand is allowed to bind to the active site of the target molecule within the ligand conformational space.^17^ The system’s total energy is calculated using the ligand’s internal and external variation movements.

The surface features of the ligand and the target molecule are utilized in the shape complementarity approach, and the complementarity of the ligand and the target molecule surfaces depend on the shape matching illustration that is used in searching the complementary pocket for ligand docking on the molecular surface of the target molecule.^17^

### 3.1 Molecularly Imprinted Polymers (MIPs) as Biomimetic Ligands

MIPs, known as the plastic antibodies or tailor-made receptors, got the attention of researchers during the mid-1980s,^18^ and today, these plastic antibodies are employed in diverse applications, including biosensing applications.^19^

**Figure 2:**
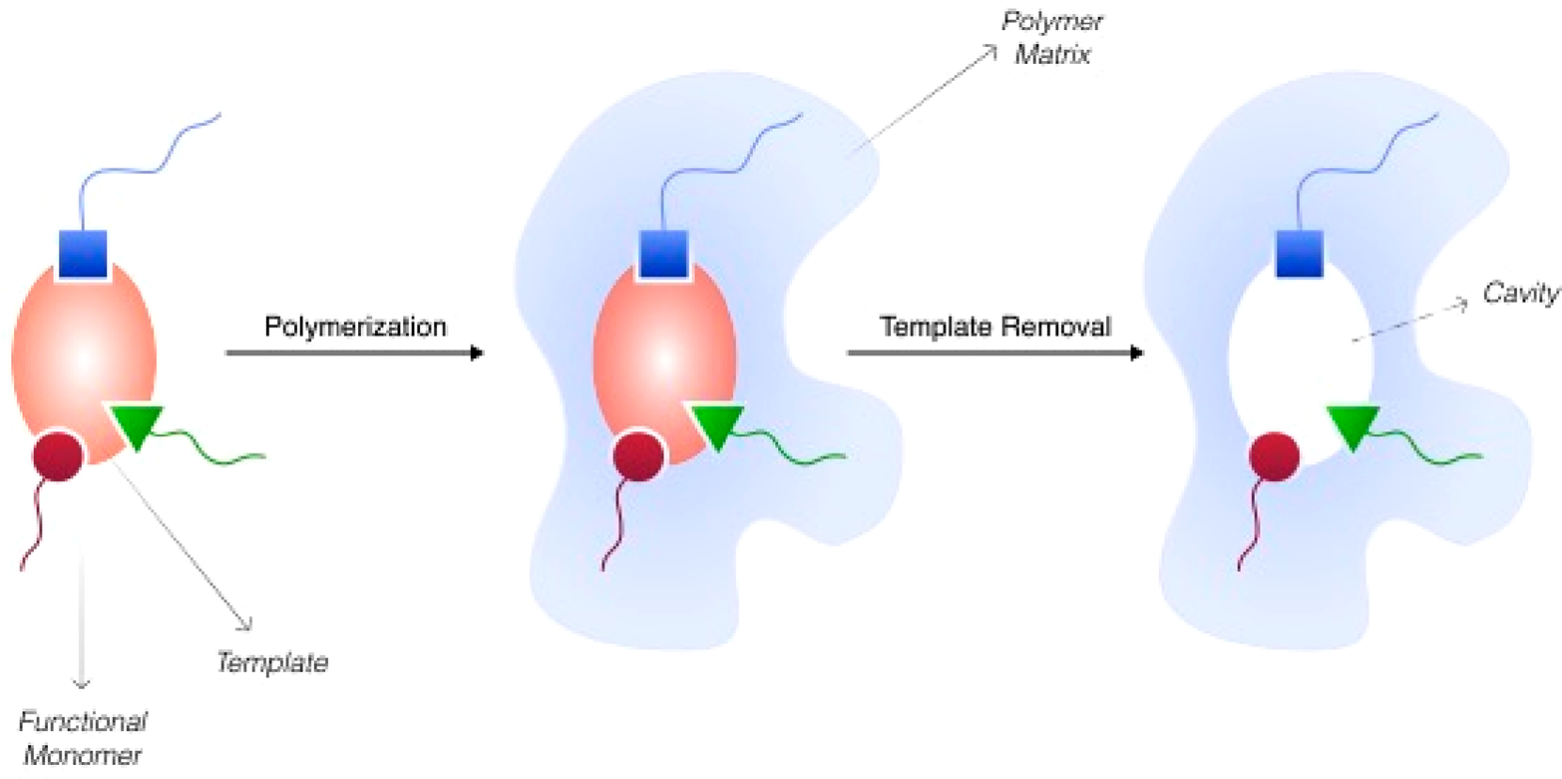
Schematic representation of MIPs.

Before creating the artificial receptors (Figure 2), the functional monomers are formed around the template molecule using covalent, noncovalent, or semicovalent interactions. After that, the polymerization occurred by using a proper cross-linker and initiator in a polymer matrix. Finally, the template molecule is removed from the polymer matrix to create the specific binding cavities that recognize it via its shape, size, and 3D structure.

The functional monomer(s) and a template molecule are crucial in designing the specific recognition cavities; however, monomer–monomer, monomer-template, or solvent-template interactions can also affect the molecular and physical characteristics of the specific recognition cavities.^20^ Therefore, the knowledge of the prepolymerization complex in detail is a significant factor in designing the appropriate MIPs, and the computational methods provide much greater detail about the prepolymerization complex and allow one to characterize the polymer-ligand interactions when compared to the classical thermodynamic models.^21−24^

The first study of this section was reported by Han et al.,^25^ who designed MIPs with the aid of molecular modeling (HyperChem software) to detect sulfonylurea herbicides (SUs) from the food samples, and the analysis of the target herbicide, metsulfuron-methyl (MSM), was carried out using high-performance liquid chromatography-tandem mass spectrometry (HPLC-MS/MS). Before the experimental studies, the target pesticide, MSM, was chosen with the aid of the principal component analysis (PCA) among the other eight SUs. Afterward, trifluoromethyl acrylic acid (TFMMA) was chosen as a functional monomer according to the binding energies, and the template/monomer ratio was optimized at 1:4. The experimental studies showed that the prepared MIPs could recognize SUs with high selectivity and a low matrix effect compared with commercial solid-phase extraction (SPE) columns.

**Figure 3:**
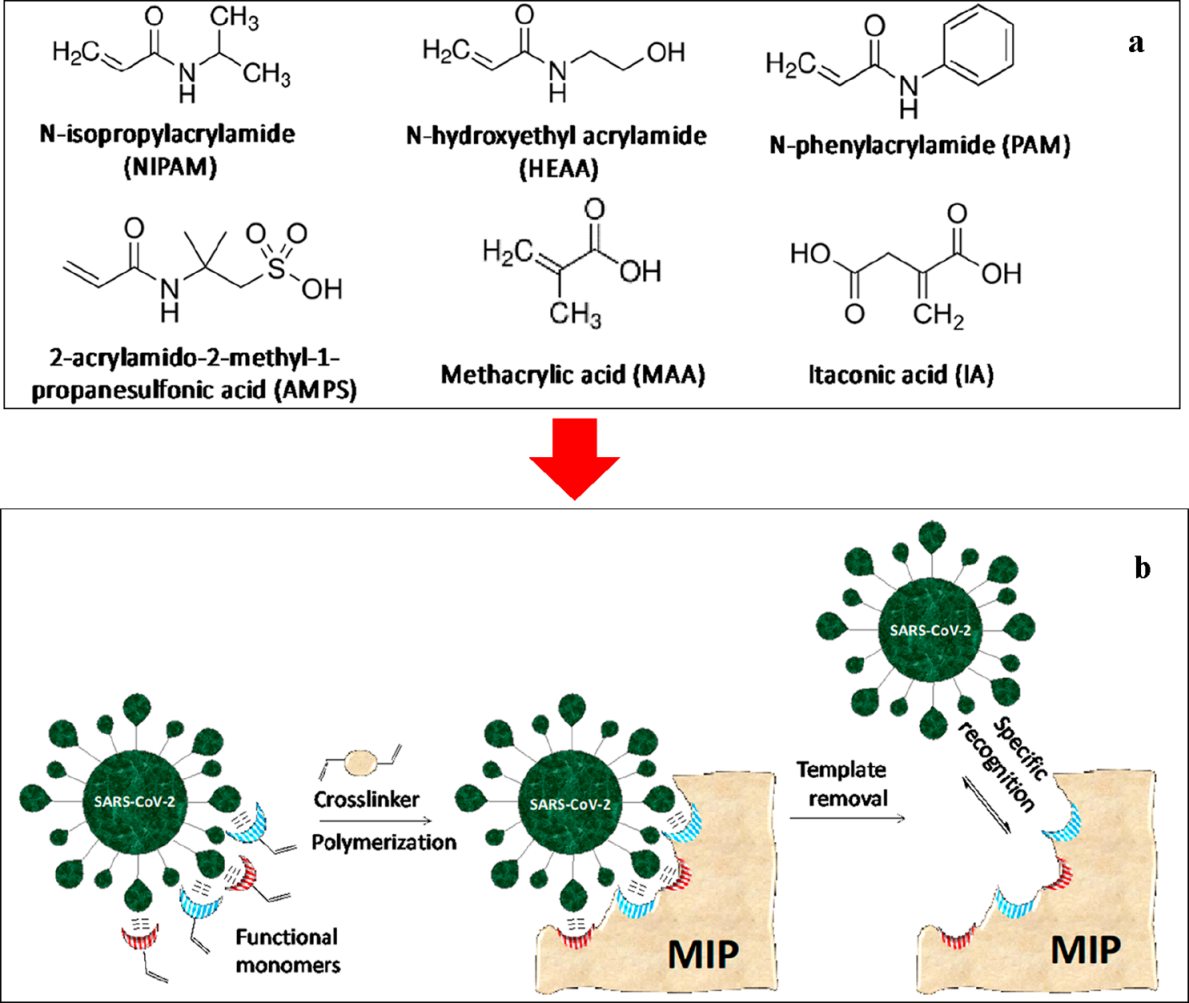
(a) Structure of functional monomers and (b) schematic representation of MIP against SARS-CoV-2. Reprinted with the permission from ref (26). Copyright 2021 Elsevier B.V.

Another study was reported by Cubuk et al.,^26^ who designed MIPs by using computational analyses [sequence analysis, molecular docking, and molecular dynamics (MD) simulation] for the detection of COVID-19 (Figure 3). During the biomimetic ligand evaluation, six different monomers [*N*-isopropylacrylamide (NIPAM), *N*-hydroxyethyl acrylamide (HEAA), *N*-phenyl acrylamide (PAM), 2-acrylamido-2-methyl-1-propanesulfonic acid (AMPS), methacrylic acid (MAA), and itaconic acid (IA)] were chosen, and their interactions of the five different regions SNNLDSKVG, LYRLFRKSNLK, TEIYQAGST, NGVEGF, and QSYGFQPTNGV sequences of SARS-CoV-2 receptor binding domains (SARS-CoV-2 RBDs) were investigated. MIPs were prepared on the TEIYQAGST region using AMPS and IA monomers through hydrogen bonds and hydrophobic interactions.

3,4-Methylenedioxymethamphetamine (MDMA), known as ecstasy, stimulates the central nervous system and increases the sociability and energy-boosting of humans; however, MDMA has some adverse effects depending on the amount of use and administration route.^27^ Sales and Ramalho^27^ prepared MIPs by optimizing the various parameters, including the template/monomer ratios, suitable solvents, and the cross-linker. For that purpose, various (11) functional monomers, six solvents, and three cross-linkers were investigated for optimization studies. Eleven competitor molecules were used to test the selectivity of the MIPs. According to the molecular electrostatic potential (MEP) map results, the monomers acrylic acid (AA), itaconic acid (IA), methyl methacrylate (MAA), and trifluoro methacrylate (TFMA) were the appropriate functional monomers for designing MIPs; however, the complex between IA and MDMA was more stable because of the stronger hydrogen bonds among the other five functional monomers. Acetone was selected as a suitable solvent according to its interaction energy variation (Δ*E*) value, and trimethylolpropane trimethacrylate (TRIM) and ethylene glycol dimethacrylate (EGDMA) were appropriate cross-linking agents because of their theoretical calculation results.

**Figure 4:**
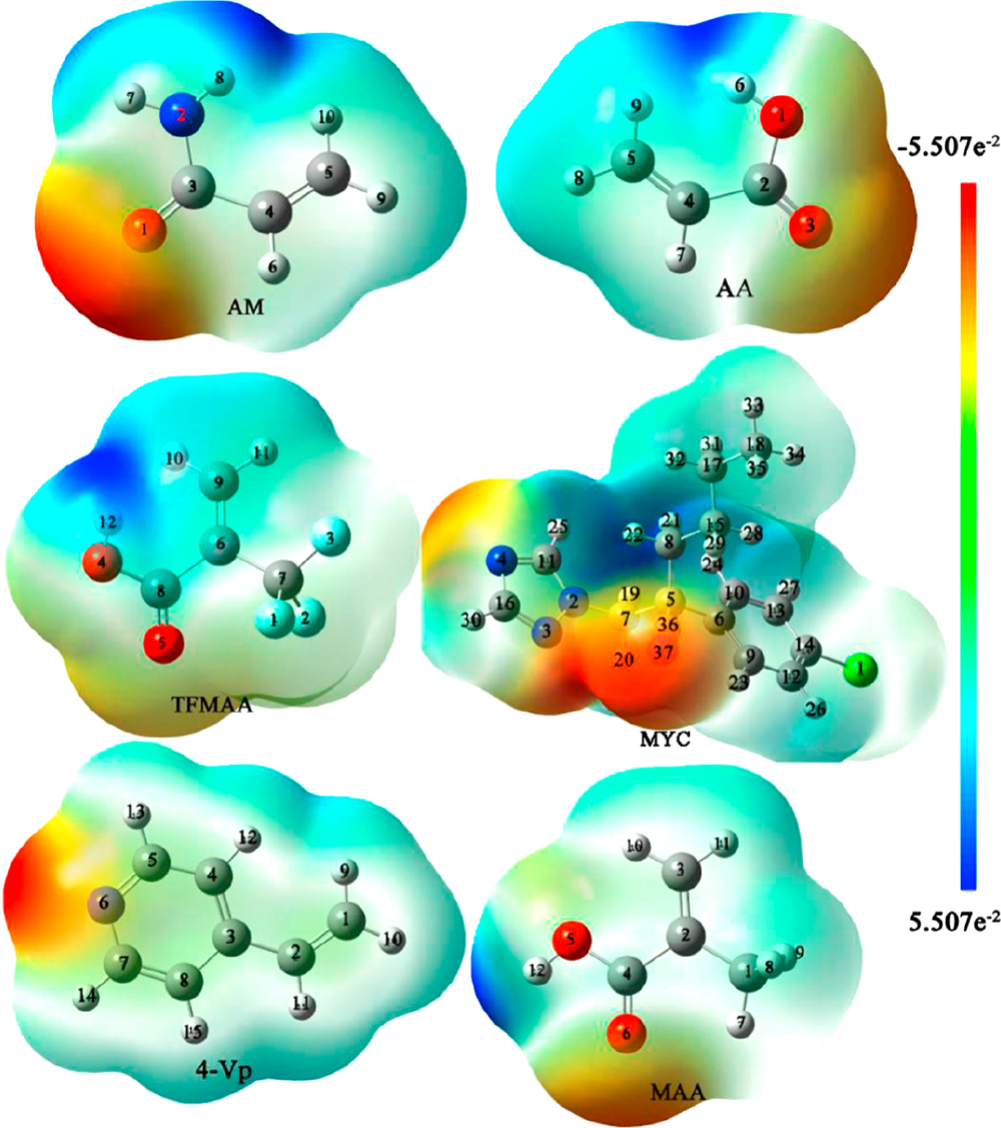
Molecular electrostatic potential (MEP) of the target molecule, MYC, and the functional monomers. Reprinted with permisson from ref (28). Copyright 2021 Elsevier.

Myclobutanil (MYC) is a broad-spectrum fungicide pesticide that remains in plants for a long time. Its residual can damage humans and animals by causing various cancer types and tumors.^28^ Li et al.^28^ used density functional theory (DFT) to prepare MYC imprinted nanoparticles (MYC-MINs). They tried to estimate a suitable functional monomer, a solvent, and a prepolymerization temperature with the calculation results. For that purpose, five different monomers, methacrylic acid (MAA), trifluoromethyl acrylic acid (TFMAA), acrylic acid (AA), acrylamide (AM), and 4-vinylpyridine (4-VP), were analyzed (Figure 4), and the appropriate monomer was chosen according to the binding energy results. After optimizing the suitable MYC-functional monomer complex, the template-monomer complex was simulated using seven kinds of solvents to predict the suitable solvent. The experimental studies were carried out using ultrahigh-performance liquid chromatography (UHPLC). The experimental results showed that TFMMA and toluene were suitable functional monomers and solvents, respectively, and the prepolymerization temperature was determined at 30 °C. The selective adsorbent with a 134.26 nm diameter was fabricated using a 1:4:20 template:monomer:cross-linker (EGDMA) ratio, and its adsorption capacity and adsorption equilibrium time were identified as 4.78 mg/g and 90 min, respectively.

The other applications of MIPs are shown in Table 1.

**Table 1:** Other Applications of MIPs use Computational Approaches^a^

functional monomer	template	cross-linker	solvent	ref
trifluoromethacrylic acid	norfloxacin	TRIM	toluene	(29)
pyrrole	closental	–	ethanol	(30)
APTES	bisphenol A	EGDMA	acetonitrile	(31)
methacrylamide	bilobalide	TMPTA	acetonitrile	(32)
4-VP and methaacryclic acid	acetaminophen	–	tetrahydrofuran	(33)
methacrylamide	ginkgolide B	EGDMA	acetonitrile	(34)
pABA-*co*-DDS	tetradifon	–	water and acetonitrile	(35)
acylamide	deltamethin	EGDMA	*N*-hexane	(36)
para amino benzoic acid	diosgenin	–	phosphate buffer	(37)
thiosermibarbazone monomers	catechin	EGDMA	acetone/acetonitrile	(38)
arginine	theophylline	–		(39)
acrylic acid	buprenorphine	EGDMA	DMSO	(40)
methacrylic acid	norfloxacin	EGDMA	DMSO	(41)
itaconic acid	nevothroxine	EGDMA	*N*-hexane	(42)
methylacrylamide	eactopamine	–	DMSO	(43)
methacrylic acid	levetiraetam	EGDMA	chloroform	(44)
methacrylic acid or 2- (trifluoro methacrylic acid)	atrazine	–	toluene	(45)
M-phenylenediamine	immunoglobulin G	DTPPS	ethanol	(46)
methacylamide	morphine	EGDMA	water	(47)
APTES	sulfamethoxazole	TEOS	ethanol	(48)
methacrylic acid	celecoxib	EGDMA	acetonitrile	(49)
methacrylic acid	phenol	EGDMA	toluene	(50)

a Abbreviations: APTES (3-aminopropyltriethoxysilane); 4-VP (4-vinylpyridine); pABA-co-DDS (para amino benzoic acid and 4,4-diaminodiphenyl sulfone); TRIM (trihydroxymethylpropyl trimethyl acrylate); TMPTA (trimethylolpropane triacrylate), DMSO (dimethyl sulfoxide), DTSSP (3,3′-dithiobis (sulfosuccinimidylpropionate), and TEOS (tetraethyl orthosilicate).

### 3.2 Other Biomimetic Ligands

Cibacron blue F3G-A was the first biomimetic triazine dye ligand used to purify yeast enzyme phosphofructokinase and a plethora of proteins, respectively.^4^ After, the importance of the sulfonate ring analogs on the dyes for the interaction and binding of proteins has paved the way for designing the new ligands and the new triazine-based affinity adsorbents.^4^ In some cases, the dye ligands could bind unrelated proteins with high affinity, thus the target protein is eluted using an affinity elution or a new dye ligand is created to overcome the selectivity problem.^51,52^

**Figure 5:**
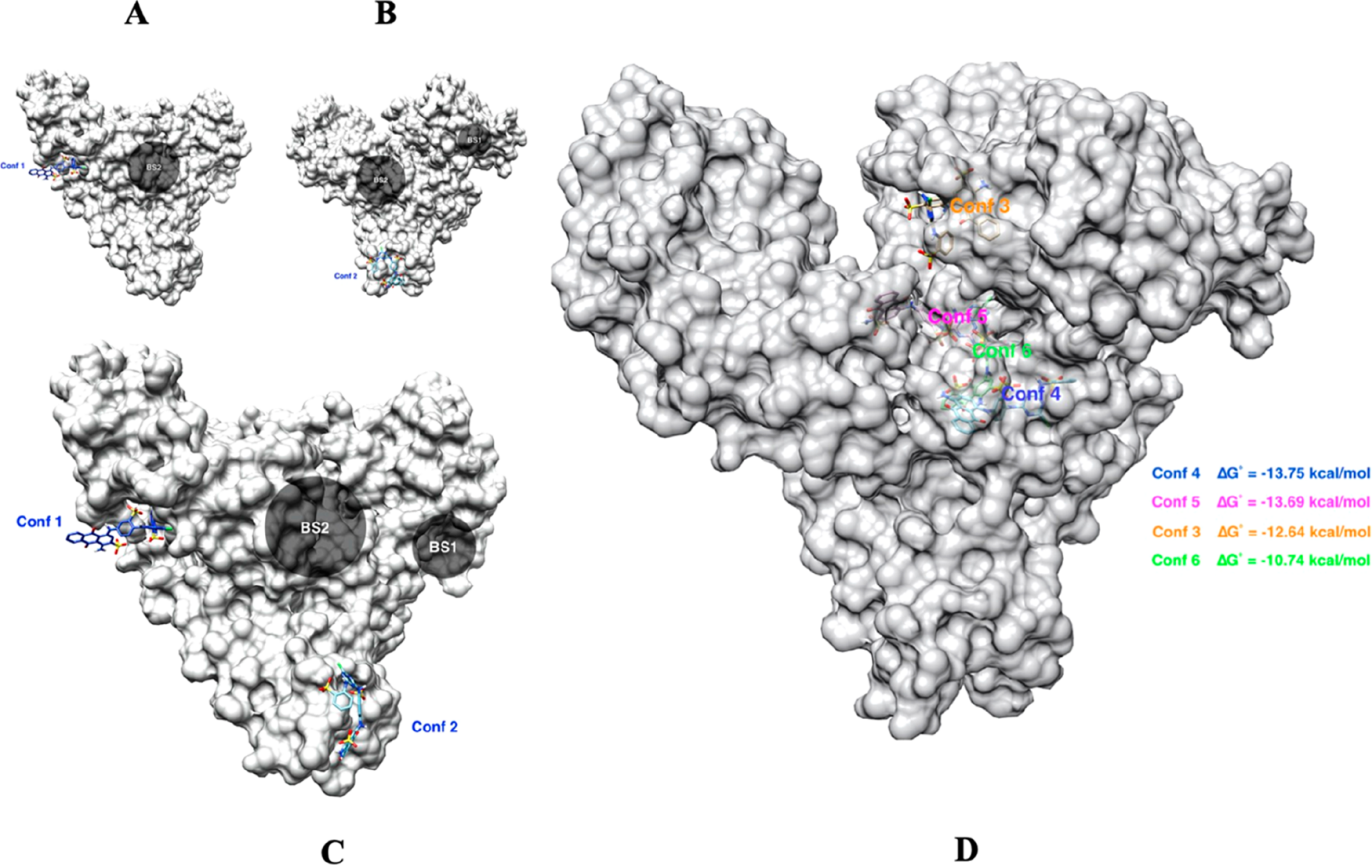
A, B, C, and D represent the six conformations of CB with HSA. Reprinted with the permission from ref (53). Copyright 2021 Elsevier.

Molecular modeling applications are used to predict the protein-ligand interactions and design the highly selective biomimetic dye ligands for the target molecules.^53^ For instance, Kılıç et al.^53^ used computational methods to investigate the appropriate complexes between CB and human serum albumin (HSA). For this purpose, the AutoDock v4.2.6 software was used, and six conformations of CB were investigated using molecular docking software to predict the binding preference of the biomimetic dye and the target protein (Figure 5).

In accordance with the molecular docking analysis, conformation 5 is the favorable ligand with the lowest binding energy among the other biomimetic ligands. The dye ligand and HSA depend on hydrophilic and hydrophobic interactions thanks to the sulfonic and anthraquinone groups, respectively. Moreover, hydrogen bonds between the anthraquinone groups and the amino acid backbone in Domain IIIA of HSA play a significant role in the interactions of the dye and the target protein.

Song et al.^54^ investigated the interaction and inhibition mechanisms of Direct Red 80 (DR80) dye and α-amylase enzyme by using multi spectra, molecular docking, thermodynamic analysis, and an enzyme activity test. In accordance with the experimental results, the skeleton structure of the α-amylase was loosened and unfolded by DR80. The increasing amount of DR80 caused a decrease in the size of α-amylase. The binding process of DR80 and α-amylase enzyme is exothermic and spontaneous, and hydrogen bonds play a significant role in the binding process. DR80 as an azo dye preferably bounds the enzyme through the surface domain A instead of the active site of the α-amylase enzyme and causes the change of enzyme structure resulting in the loss of enzyme activity.

The following research article aimed to investigate the toxic effects of the dye molecules on protein binding and hemoglobin (Hb) was chosen as a well-known model protein. Its interactions and binding conformations with the four organic dyes (fluorescein, congo red, methyl red, and methyl orange) were examined using spectroscopic techniques and molecular modeling.^54,55^ The association constants of the dyes were similar, and the dyes could bind hemoglobin with strong interactions. The azo dyes have high toxicity, but the toxic effects of fluorescein and congo red were similar. With the light of the fluorescence studies, congo red and methyl red had a single binding site for Hb, and molecular modeling showed that all dyes could bind Hb within its central cavity.

The summary of the biomimetic dye ligands and the design of triazine scaffolds using computational methods and their affinity chromatography applications are illustrated in Table 2.

**Table 2:** Summary of the Biomimetic Dye Ligands and the Design of Triazine Scaffolds Using Computational Methods and Their Affinity Chromatography Applications

dye or ligand	target molecule	purpose	method	ref
triazine dye	glutamate oxidase	purification	bioinformatic analysis	(56)
ligand 22/8	human immunoglobulin G (IgG)	purification	molecular modeling	(57)
red HE-3B	lactoferrin	analysis of the dye and protein binding sites	molecular modeling	(58)
cibacron blue 3GA	antibody 2G12	purification	molecular modeling and molecular dynamics simulation	(59)
galactosyl-mimo dye ligands	galactose dehydrogenase	purification	molecular modeling and ligand docking	(60)
rhodamine B	human serum albumin	analysis of interaction of dye and protein	molecular modeling and molecular dynamic simulations	(61)
azo dye (amaranth)	bovine serum albumin	analysis of interaction of dye and protein	molecular docking studies	(62)
allura red AC	human serum albumin	analysis of binding interaction dye and protein	molecular modeling	(63)
azo dyes	lysozyme	analysis of molecular reaction of dye and protein	molecular modeling	(64)
C.I. acid red 88	serum albumins	analysis of binding behavior of dye and proteins	molecular modelingMD	(65)

Aptamers are short, single-stranded DNA or RNA oligonucleotides that can recognize the target molecules with high affinity and selectivity depending on their unique tertiary structures.^66,67^ The interactions between the aptamers and the target proteins are favorably electrostatic forces; however, the multiple weak interactions such as hydrogen bonds, hydrophobic interactions, and shape-forming features are also of great importance for their recognition capabilities.^65^ These affinity probes possess superior features such as low cost, high affinity toward the target molecule, low dissociation constant (KD), and low immunogenicity.^66^ Furthermore, aptamers are more stable than antibodies, thanks to the robustness of the phosphodiester bond. In this regard, these antibody analogs hold great potential in various fields.^65,67^

The systematic evolution of ligands by an exponential enrichment (SELEX) technique was used to create the aptamers during the early studies of aptamer selection. This repetitive method involves incubation, binding, partitioning, and amplification steps.^68,69^ However, the whole process of SELEX takes days to months and could reach up to 15 rounds.^69^

Computational methods are essential for biological studies^70^ and are used to determine the binding sites in the enzyme, protein structure-function study, and drug-screening applications.^68^ Recently, the computational approaches aim to minimize the number of sequences in the library pool and accelerate the whole process while finding the desired aptamer sequences.^68,71^ So, *in silico* approaches based on molecular dynamics and molecular modeling can potentially develop the specific aptamer toward its specific target molecule.

Aptamer LC-18 is composed of 80 nucleotides containing two constant 20 nucleotide primers on each side and shows good binding affinity toward the lung carcinoma cells, blood plasma, and tissues.^72^ Morozov et al.^72^ designed a new truncated LC-18 (LC-18t) aptamer with the aid of computational approaches to reduce its size to increase the binding affinity of the new aptamer, and the molecular structure of LC-18t was compared with small-angle X-ray scattering (SAXS). After that, the selectivity of the new aptamer was investigated against the lung carcinoma cells. During the LC-18t, the 35 nucleotides on LC-18 were truncated according to the prediction of secondary and tertiary structures. The replacement analysis showed that the binding affinity of LC-18t was much higher than LC-18. The aptamer LC-18t can bind the lung carcinoma cells; however, it has no remarkable binding affinity toward the healthy tissues. The molecular modeling results of LC-18t were following SAXS data, and the LC-18t had the same binding sites with the long aptamer.

**Figure 6:**
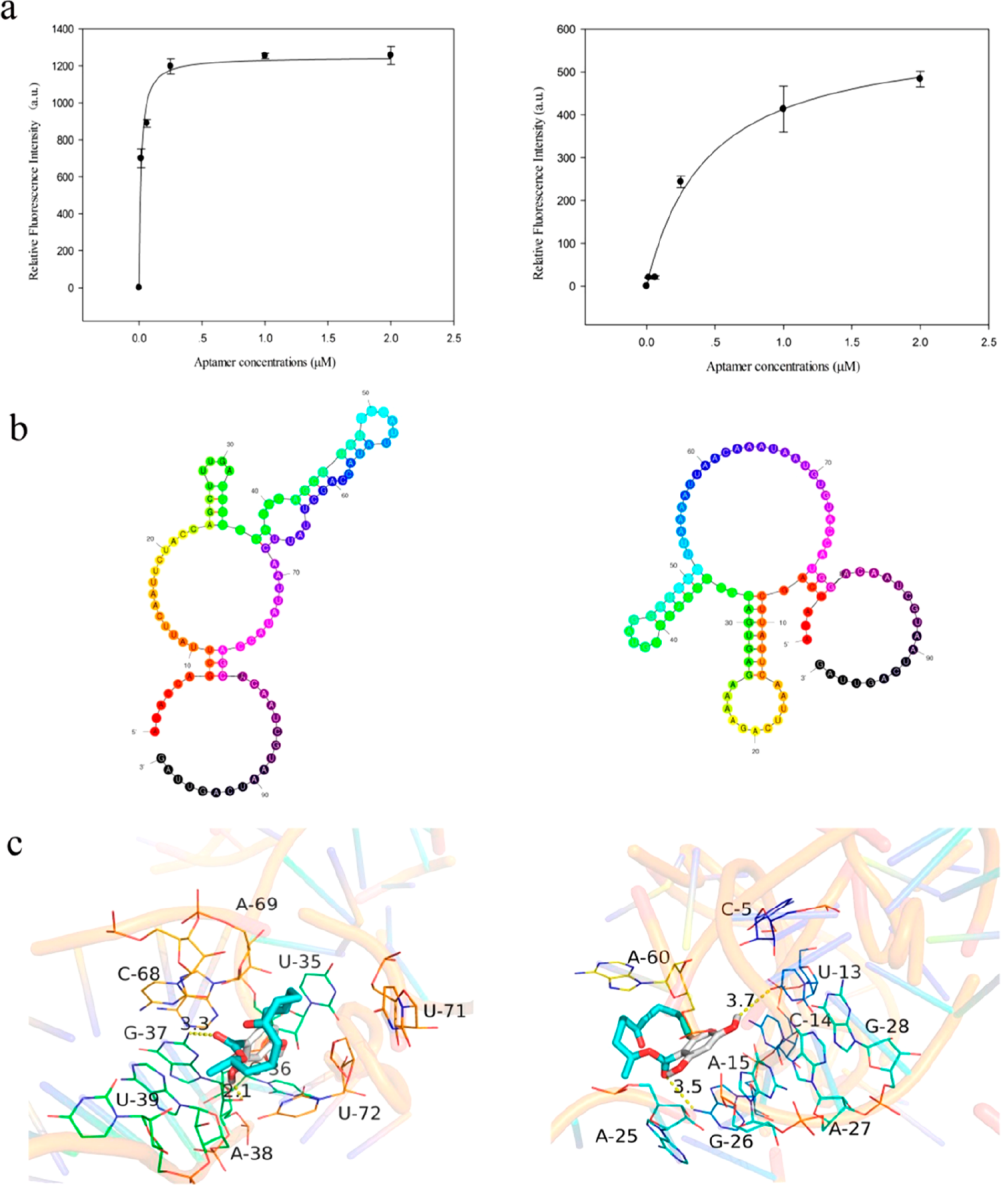
(a) Characterization of aptamers, (b) 2D structures prediction of aptamers with the aid of the mfold and 3dRNA-V2.0 online tools, and (c) binding modes of aptamers and ZEN molecule via molecular docking. Reprinted from ref (73). Copyright 2018 ACS.

In the following study, an aptasensor was developed to detect zearalenone (ZEN), a nonsteroidal estrogenic mycotoxin with some side effects on animal health.^73^ For this aim, the aptamers were developed with the SELEX process, and the binding modes of the aptamer and ZEN were investigated with molecular docking (Figure 6). After optimizing the selected aptamers, label-free ZEN detection dependent on the color change of the solution was carried out using gold nanoparticles.

The noncovalent bonds in the binding sites of the developed aptamers and ZEN molecule played a crucial role in the recognition process, and the experimental results revealed that the developed aptasensor could detect ZEN in animal feeds.

Angiopoietin-2 (Ang2) plays a significant role in regulating vascular stability and is expressed only at sites of angiogenesis; therefore, the monitoring of Ang2 is of great importance for clinical studies.^74^ Hu et al.^74^ used a computational approach (ZDOCK and ZRANK algorithms) to design an RNA-based surface plasmon resonance (SPR) aptasensor for screening Ang2. The 15 Ang2 aptamers were analyzed. In accordance with the ZRANK scores, the aptamer, Seq1 (with the highest binding affinity), and the aptamer, Seq16 (with the lowest binding affinity), were considered appropriate and control aptamer, respectively. Furthermore, three aptamers were mutated at different positions to compare the binding affinity of the original aptamers toward Ang2, and the binding kinetics of the mutated and the original aptamers were investigated with SPR signals. The aptamer Seq1 with the highest *k*_a_ value could generate more SPR signals than the other aptamers, and the simulation results of Seq1 and Seq16 were in accordance with the experimental findings. However, the expected computational results of the mutant aptamers were not in agreement with the experimental results, and the researchers suggested that the experimental conditions such as the pH of the solution or the ionic strength may influence the actual interactions of the mutant aptamers, and Ang2 could result in finding different experimental results than the simulation results.

In Table 3, we summarized the design of aptamers with the aid of computational approaches and their usabilities for different applications.

**Table 3:** Some of the Aptamers and Their Applications

aptamer	target	method	purpose	ref
APTSTX-1	saxitocin	molecular dynamics	sensing	(75)
Z3IN	zearalenone	computational docking simulation	sensing	(76)
P-30	patulin	molecular docking and circular dichroism	detection	(77)
AOT1 conjugated gold nanoparticles	oxytetracycline	molecular docking and circular dichroism spectroscopy	detection	(78)
MApta^pro^-IR1	SARS-CoV-2 M^pro^ enzyme	molecular docking and molecular dynamic simulation	to develop a therapeutic drug for the COVID-19 disease	(79)
the conjugation of aptamer and single-walled carbon nanotubes	prostate-specific antigen (PSA)	molecular dynamic simulations	to understand the interaction mechanism and design an aptasensor	(80)
Tro4apt	cardiac troponin I	docking and molecular dynamics	screening and sensor development	(81)
F20	aflatoxin B_1_	combination of *in silico* maturation and molecular docking	sensing	(82)
AT11	nucleolin	*in silico* molecular docking simulations	to investigate the activity of the aptamer toward the target	(83)
FLC112	angiotensin II	mocking simulation	to investigate the interactions of the aptamer and the target	(84)
DF20	diazinon	docking and molecular dynamic simulation	sensing	(85)
51A1	cytochrome p450	molecular docking and molecular dynamics	to design the aptamer	(86)
RNA aptamer	flavin	molecular dynamics simulations	to design the sensor	(87)
P-18S2	palytoxin	molecular docking and molecular dynamic simulations	to understand the binding mechanism of the aptamer and the target	(88)
RBA	retinol binding protein 4	molecular dynamics simulations	to understand the binding mechanism of the aptamer and the target	(89)
RNA aptamer	cell surface protein of *Streptococcus agalactiae*	molecular docking	to design and optimize the aptamer	(90)
WGQWPYHC	targeting translationally controlled tumor protein (TCTP)	molecular docking studies and bioinformatics	to investigate the interactions between the aptamer and the protein	(91)

## 4 Conclusion

Observing life via science and mimicking the biological systems has promoted humanity to make innovative products and nanomachines to improve quality of life. Furthermore, biomimetics opened up new avenues in life sciences and provides an understanding of biological processes.

Molecular recognition such as antigen-antibody, enzyme-substrate, and protein-ligand interactions is the center of biological processes. The researchers mimic molecular recognition to design synthetic systems for various fields, e.g., such as biosensing platforms and drug discovery studies, and purification of biomolecules.

Affinity chromatography is a powerful separation method of biomolecules from crude samples based on molecular recognition, and the selection of an affinity ligand is the success of affinity chromatography.

Biomimetic affinity ligands can mimic the structure and binding sites of the bioligands; these synthetic ligands meet the requirements of the bioligands and are favorable candidates for affinity chromatography and its diverse applications.

In recent years, computational approaches, e.g., machine learning and deep learning algorithms, provide a way to predict the ligand binding sites of the protein, and identifying these binding sites gives some pieces of information about the intermolecular interactions.^92^ Furthermore, the efficiency and accuracy of prediction of ligand binding sites could be improved by combining computational approaches and experimental studies.^92^ So, computational approaches play a significant role in developing biomimetic affinity ligands.

This review introduced the commonly used biomimetic affinity ligands and their developing strategies and highlighted their usability potential for affinity chromatography applications.

In our opinion, in the future, the development of computational approaches like deep learning and machine learning has accelerated the design of new biomimetic ligands and offers new opportunities for life sciences.
